# A Qualitative Systematic Review of the Role of Entrustment in Pre‐Registration Healthcare Practice‐Based Learning: Implications for Dietetics Education

**DOI:** 10.1111/jhn.70287

**Published:** 2026-06-10

**Authors:** Ruth Boocock, Cecile Jones, Amelia A. Lake

**Affiliations:** ^1^ School of Health & Life Sciences, Centre for Population Health and Healthcare Teesside University England UK

**Keywords:** capability development, dietetic education, entrustment, health professions education, practice‐based learning, qualitative systematic review, workplace‐based assessment

## Abstract

This qualitative systematic review explored how entrustment is understood, experienced and enacted within pre‐registration practice‐based learning (PBL) across international healthcare education, with a specific focus on implications for the UK dietetic profession. Fifteen qualitative and mixed methods studies were synthesised using thematic synthesis, identifying how entrustment shapes learner participation, supervision, and capability development. The review found that entrustment functions as a ‘credible developmental space’ that enables learners to engage in authentic workplace activities with supported autonomy, while also revealing how workplace pressures and local contextual factors influence entrustment decisions. Feedback emerged as the central mechanism through which learners make sense of supervision levels, understand expectations, and professionally develop; without high quality dialogic feedback, entrustment risks becoming a procedural rather than educational process. Entrustment was shown to be inherently relational and co‐constructed, shaped by trust, supervisor familiarity, contextual constraints, perceived risk, and the quality of supervisory relationships. These findings challenge overly technical or checklist based interpretations of entrustment and highlight the need for assessment approaches that prioritise narrative feedback, shared sensemaking and contextual awareness. For UK dietetics, an entrustment informed approach offers a defensible, capability‐aligned way to structure PBL across diverse settings, particularly as the profession implements a national common assessment tool. A proportionate, light touch approach, using a small number of meaningful EPA aligned tasks supported by succinct narrative feedback, may enhance developmental value without increasing burdens on practice educators. Entrustment has the potential to strengthen fairness, consistency, and capability development in dietetic PBL when implemented in a relational, context sensitive manner. These insights have relevance globally, providing a conceptual foundation for international dietetics and wider health professions to strengthen supervision and assessment in diverse PBL contexts.

## Summary

- Entrustment provides a structured way to support learner capability development, helping practice educators make proportionate, feed‐forward supervision decisions that focus on growth rather than minimum competency checklists.
- High quality, dialogic narrative feedback is essential for learners to interpret supervision levels, understand expectations, and progress safely; supervision ratings alone are insufficient without meaningful feedback conversations.
- Entrustment decisions are relational and context dependent, influenced by trust, workload, familiarity with the learner and workplace pressures, highlighting the need for practical educator preparation, shared understanding, and simple, streamlined assessment tools.

## 1 Introduction

Practice‐based learning (PBL) in pre‐registration healthcare education refers to the structured learning that takes place in real or simulated clinical or workplace environments, bridging the gap between classroom learning and professional practice, and is often termed placements [1]. Here, ‘pre‐registration’ refers to education programmes leading to eligibility for professional registration. These opportunities enable learners (students, apprentices, or trainees) to apply, develop and consolidate knowledge, skills and behaviours under supervision. PBL is a core requirement for all UK pre‐registration healthcare degrees regulated by bodies such as the Health and Care Professions Council (HCPC), the Nursing and Midwifery Council (NMC) and the General Medical Council (GMC). While each regulator defines PBL slightly differently, common principles are consistent across professions and usually involve assessment of learner performance.

Across UK healthcare education, regulators and professional bodies vary in how they emphasise competency and capability. These terms appear similar but describe fundamentally different ways of understanding professional performance. A competency is typically defined as a specific, observable skill or behaviour that can be measured through direct assessment [2]. In practice, this means demonstrating that a learner can carry out a clearly defined task to an acceptable standard, for example, taking a patient's blood pressure, completing a nutritional assessment. Competencies are often checklist‐like breaking down professional roles into discrete tasks that can be assessed one at a time, usually in controlled or predictable circumstances. By contrast, a capability describes something more holistic, a learner's ability to draw together and flexibly apply their knowledge, skills and behaviours when faced with complex, uncertain, or unfamiliar situations [3]. While competencies show whether someone can perform a task, capability reflects whether they can adapt, make judgements, and respond appropriately when the situation is complex or unpredictable. For example, when a patient deteriorates unexpectedly, when priorities must be balanced, or when working within a new team or setting.

UK healthcare regulators illustrate these differences in emphasis. The HCPC uses a blend of competency‐like standards, alongside broader expectations that reflect adaptive capability [4]. The NMC places stronger emphasis on competencies, describing the specific tasks and behaviours expected of a newly registered nurse, with some recognition of adaptability embedded into its standards [5]. The GMC takes the most capability‐orientated stance, intentionally preparing new doctors to manage uncertainty, complexity, and rapid change in modern healthcare environments [6].

Competency‐based healthcare education tends to rely on demonstrating discrete tasks, often assessed at the ‘does’ level of Miller's pyramid, which focuses on what learners can show in a given moment [7]. To address developmental progression, some healthcare programmes draw on frameworks such as the Dreyfus model which maps a learner's journey from novice to expert over time [8]. However, the Dreyfus stages can be difficult to apply consistently because they do not always account for how performance varies within context, team dynamics, emotional load, or workplace pressures. Competency‐based assessments are useful for confirming minimum standards of performance [5]. However, they often provide only isolated snapshots of observable behaviour and therefore fail to show whether learners can adapt across contexts, manage unpredictability, or work with autonomy, offering limited insight into their development in real‐world practice [9].

Internationally, Entrustable Professional Activities (EPAs) have become a key mechanism for operationalising competency‐based healthcare education. Developed in the Netherlands in 2005, EPAs are now widely used across countries such as Canada, the United States, Australia and New Zealand [10]. They are defined units of professional practice that a learner may undertake with decreasing supervision once deemed ready by their practice educator (supervisor, assessor, or mentor). Although developed within medical education, EPAs have since expanded across multiple healthcare professions, including nursing, pharmacy, veterinary medicine, physiotherapy, and dentistry [11, 12]. While this literature is well established across health professions, its application within dietetics remains less developed. A recent systematic review identified six studies globally examining EPAs in nutrition and dietetics education, highlighting the early stage of development of this field [13]. Existing work, largely led by Bramley and colleagues, suggests that EPAs can support translation of competency frameworks into observable practice, promote reflective learning, and facilitate feedback and dialogue between learners and practice educators [14, 15]. However, this literature is predominantly focused on tool development within single institutions, with comparatively limited exploration of the relational processes underpinning assessment. Consequently, there remains a need to better understand how entrustment shapes discussions of uncertainty within PBL. In the UK, EPAs are emerging within pre‐registration medical education and selected postgraduate specialties [16], with early uptake also visible in pharmacy education [17].

A defining feature of EPAs is the use of entrustment‐based decision making. Learners progress through several levels of supervision, from observation to independent practice [18]. This approach requires practice educators to judge not only what a learner did in a specific encounter but what level of supervision they can safely manage in future practice. Entrustment decisions are influenced by multiple factors including task complexity and risk, the PBL setting, the practice educator's familiarity with the learner, and their inclination to trust [18, 19]. Summative entrustment‐based decisions are typically made by programme‐level committees and rely on aggregated holistic narrative evidence, rather than isolated observations, reflecting the need for sufficient trust to authorise progression [20]. While EPAs aim to support holistic assessment, they rely on context‐dependent, holistic judgements about supervision and autonomy, which may introduce variability in entrustment decisions.

Mason's framework of ‘safe uncertainty’ offers a helpful lens for practice educators by providing a ‘mindset’ and a teachable construct widely used in systemic practice [21, 22]. It acknowledges that uncertainty is inherent in clinical work and encourages healthcare professionals to uphold safety while resisting premature or overly confident judgements. Safe uncertainty can facilitate honest discussions about uncertainty within supervisory relationships, supporting shared reflection and sensemaking [23]. Entrustment naturally aligns with this stance by calibrating supervision as learners manage increasing complexity.

In the UK, PBL is expanding across clinical and non‐patient facing settings to address workforce pressures and build sustainable, inclusive PBL capacity [24]. This diversification reflects contemporary health and social care models, and the need to embed the four pillars of practice (clinical, education, research and leadership) early in learner development to strengthen professional capability and enhance graduate employability and retention [25]. As EPAs are task‐focused and transferable, they have the potential to support learning across varied PBL landscapes and can be clustered to reflect pillar‐specific activities [2].

Taking a systems approach to PBL, standardised PBL assessment tools are widely used internationally, with scope ranging from single higher education institutions to national frameworks. In the UK, pre‐registration dietetic education is delivered through accredited programmes aligned to HCPC standards and the BDA curriculum framework. PBL is a core component, with learners typically completing a minimum of 1000 h across diverse settings, integrated with academic learning, making PBL assessment central to determining readiness for professional registration. Common assessment tools for PBL aim to improve consistency, equity, portability across PBL settings, efficiency, and quality assurance [26]. The recent soft launch of a UK‐wide dietetic common assessment tool in 2025 signals a profession‐wide move toward more standardised and capability‐aligned assessment. These aims align with national drivers to ensure that PBL functions effectively across all settings. This systematic review therefore asks, ‘How is entrustment conceptualised and operationalised in pre‐registration healthcare PBL, and what are the implications for the UK dietetic profession?’

### 1.1 Rationale

Despite the rapid international growth of EPAs and entrustment, existing evidence on how entrustment is understood, experienced, and enacted during pre‐registration PBL remains fragmented, largely centred on medical education, and shaped by diverse international contexts. As UK healthcare professions adopt capability based frameworks, expand PBL into a wider range of settings, and implement common assessment tools, a clearer understanding of the relational, contextual, and educational dynamics of entrustment is urgently needed. A qualitative systematic review is therefore timely to support professions, such as dietetics, as they embed capability based, cross setting PBL and seek defensible approaches to judging readiness for safe, increasingly independent practice in complex, changing and unpredictable environments.

### 1.2 Objectives

− How do learners and practice educators describe and experience entrustment during pre‐registration PBL?
− What educational functions does entrustment serve, and what unintended consequences are reported?
− What contextual and relational factors shape entrustment‐based decisions?
− How might entrustment support capability development under ‘safe uncertainty’, helping learners navigate complexity without premature certainty?
− What are the implications for UK dietetics, particularly regarding capability based frameworks, diverse PBL settings, and national common assessment approaches?

### 1.3 Methodology

This systematic review was conducted in line with Joanna Briggs Institute (JBI) guidance for qualitative evidence synthesis, incorporating systematic searching, critical appraisal, and reflexive analysis [27]. The review was registered with PROSPERO (International Prospective Register of Systematic Reviews; CRD420251177659) and is reported in accordance with the Preferred Reporting Items for Systematic Reviews and Meta‐Analyses (PRISMA) 2020 guidelines to ensure transparent reporting [28].

### 1.4 Eligibility Criteria

The PEO framework (Population, Exposure, Outcome) was used to develop the research question and guide the inclusion criteria (Table 1) [29]. The review included both qualitative studies and mixed methods studies where the qualitative data could be clearly isolated and extracted. Inclusion criteria comprised empirical studies examining entrustment or EPAs within pre‐registration or equivalent pre‐entry level healthcare education across professions. Studies from medicine, nursing and allied health were included to reflect the interdisciplinary nature of PBL. No year limiters were used. International studies were included allowing exploration of different pre‐registration healthcare education systems, existing entrustment‐based assessment processes, and reported facilitators and challenges to entrustment‐based decision making during PBL. Studies were limited to the English language.

**Table 1:** Eligibility criteria using the PEO framework.

	Inclusion criteria
**Population (P)**	Healthcare/Medical/Allied Health Education Pre‐registration Practice Educators, Supervisor, Mentor, Learners, Students, Apprentice, Undergraduate, Postgraduate Academics, Clinical/Practice Teachers
**Exposure (E)**	Entrustment‐based decision making/supervision/assessment Entrustable Professional Activities (EPAs)
**Outcome (O)**	User experience/perceptions/views/attitudes/beliefs/opinions/perspectives/thoughts/judgements/expectations/behaviours Impact/effect/consequences/implication on learner performance Readiness for practice Implementation

### 1.5 Information Sources

Five databases were searched (MEDLINE, Embase, CINAHL, Education Research Complete, and APA PsycINFO) on 10/09/2025. Grey literature (Conference repositories, ProQuest thesis and dissertation database) was searched to minimise publication bias [30]. Conference abstracts were screened for relevance but excluded from final analysis due to insufficient methodological detail. Pre‐print servers were not searched. The reference lists of all included full‐text published studies were searched to identify additional eligible studies not retrieved through grey literature searches. Reviews were excluded, although their reference lists were screened. Forward citation searching was also used to identify articles citing the studies included in this review.

### 1.6 Search Strategy

The search strategy was developed and refined by an academic librarian and the reviewer (RB). Search terms were intentionally broad to capture relevant literature across healthcare professions, and tailored to the databases. The full search strategies, including keywords, medical subject headings (MeSH), descriptors, and Boolean operators may be found in Appendix 1. Filters were applied to limit the studies to English language publications.

### 1.7 Selection Process

Stage one: All search results were imported into Endnote 2025 (Clarivate Analytics), a reference management software used to collect, organise, and cite research sources. This tool was used to deduplicate the studies and to support a manual screening process. The reviewer (RB) independently screened all titles and abstracts against the eligibility criteria outlined in Table 1. To ensure consistency and minimise selection bias, the reviewer (CJ) independently looked for inconsistencies in screening decisions, adhering to the PRISMA framework for performing systematic reviews [28]. Discrepancies were resolved through discussion. Where a consensus could not be reached, the study was included in stage two to avoid premature exclusion.

Stage two**:** The full texts of studies included after stage one were independently sifted against the inclusion criteria by the reviewer (RB) and checked by the reviewer (CJ). This process was iterative, with discussions helping to ensure consistency in how the inclusion and exclusion criteria were applied across studies. Backward and forward citation searching was completed by the reviewer (RB).

### 1.8 Data Collection Process

A customised data extraction tool was developed in Microsoft Excel and piloted, to aid data extraction from all included studies. Study characteristics including publication year, study design, context (country, health profession, learner type) as well as first order constructs (participant quotes including academic, practice educator and learner), and second order interpretations (concepts and themes) were extracted independently by the reviewer (RB) and checked by the reviewer (CJ).

### 1.9 Risk of Bias

The JBI tool was used to assess the methodological strength and limitations of the included qualitative studies [31]. The qualitative components of mixed methods studies were assessed using the Mixed Methods Appraisal Tool (MMAT, 2018 version) [32, 33]. The quality of the included studies was independently reviewed by the reviewer (RB) and checked by the reviewer (CJ). Key quality indicators and methodological limitations, identified during the quality appraisal, were used to better understand the included studies.

### 1.10 Data Synthesis

Thematic synthesis was used to analyse and synthesise the extracted data inductively. Thematic synthesis included three stages, (i) coding the text, (ii) developing the ‘descriptive themes’, and (iii) generating the ‘analytical themes’ [34].

Stage one: The data within each included study was analysed ‘line‐by‐line’ and a code assigned to quotes and important concepts and themes within the data.

Stage two: The codes were grouped into broad topics and patterns that summarised the full dataset (all included studies). These emerging descriptive themes remain ‘close’ to the included studies.

Stage three: Analytical themes were developed. These themes represent deeper level interpretations which go beyond the included studies and offer new insights and connections relevant to the research question. All coding and theme development was completed using Excel by the reviewer (RB) and checked by the reviewer (CJ).

Across data extraction and data synthesis, discrepancies between reviewers were resolved through discussion. When consensus was not achieved, a third reviewer (AL) was consulted. Detailed data extraction and regularly referring to the original studies ensured primary data was not lost during the analysis and synthesis process.

Researcher reflexivity and a collaborative approach to theme development raised awareness of the researchers' own biases and potential influence on the systematic review. The reviewers are UK registered dietitians working in research and academia. Reviewer (RB) led on the development of a dietetic common assessment tool for use in UK PBL. Their analytic approach is grounded in small q positivist processes, acknowledging the use of systematic, structured procedures while maintaining sensitivity to the interpretive nature of qualitative data. Thematic synthesis enables the integration of findings across heterogeneous studies and supports the development of actionable, analytically‐derived themes [35].

## 2 Results

### 2.1 Study Selection

**Figure 1:**
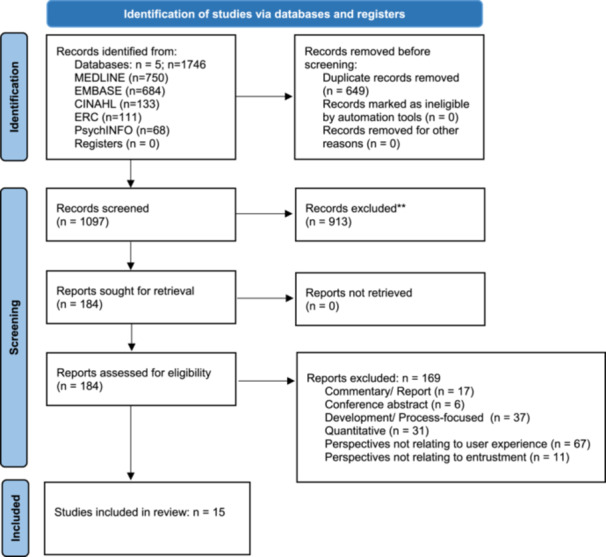
PRISMA flow chart.

Studies (*n* = 1097) were identified via the databases after removal of duplicates. Following stage one screening, 184 studies were retrieved, with 15 assessed as eligible for inclusion in this review (Figure 1). A list of excluded studies, with reason, was kept. The studies were examined to clarify profession‐specific terminology, for example ‘resident’, and to identify whether programmes were delivered at undergraduate or postgraduate level, ensuring all included studies focused on pre‐registration healthcare professional training. Studies including supervision during residency (postgraduate trainees) were excluded. A further common reason for exclusion was that certain studies centred on developing educational processes, which fell outside the scope of this review as they did not provide qualitative participant insights into their use in practice.

### 2.2 Study Characteristics

The characteristics of the 15 included studies is summarised in Table 2. An unabridged version is located in Appendix 2. The included research was conducted across diverse international settings, spanning Europe (Germany, the Netherlands, Sweden), North America (United States, Canada), and Australia [36, 37, 38, 39, 40, 41, 42, 43, 44, 45, 46, 47, 48, 49, 50]. Eight studies were qualitative. Settings were predominantly undergraduate medical education, with several studies also involving veterinary medicine and dietetics. Participant groups included students, clinical teachers, faculty assessors, and supervisors. Sample sizes ranged from small qualitative samples (*n* = 7–32) to large survey datasets (*n* > 1,500) in mixed methods designs. Qualitative data were derived from interviews, focus groups, reflective diaries, free‐text responses within surveys and structured assessment tools (portfolios and narrative feedback), where participant perspectives could be interpreted and analysed. Analytic approaches used by the studies included inductive and deductive content analysis, thematic analysis, grounded theory techniques, and, in one study, machine learning–assisted theme identification.

**Table 2:** Characteristics of included papers.

Overview			
Papers	Study characteristics	Participant demographics	Methodology
Alexander et al, 2025 [27]	Qualitative, Germany. Exploring experiences of medical students while performing clinical tasks.	Undergraduate medicine (final year). *N* = 11 (students).	Convenience sampling. Online reflective diaries. Inductive/deductive content analysis.
Bradley et al, 2021 [28]	Qualitative, United States. Investigating how students interpret and use supervision in assessments during clinical encounters.	Undergraduate medicine. *N* = 9 (students).	Convenience sampling. Semi‐structured interviews. Content analysis.
Bramley et al, 2021 [29]	Mixed‐methods, Australia. Exploring student and supervisor perceptions of assessment.	Undergraduate dietetics. For survey: *N* = 7 (students), *N* = 8 (supervisors). For consultation: *N* = 4 (students), *N* = 11 (supervisors).	Convenience sampling. Electronic surveys (free text responses), Stakeholder consultation groups. Thematic analysis.
Bremer et al, 2025 [30]	Mixed‐methods, Netherlands. Examining professional identity formation among students during clinical rotations.	Undergraduate medicine. For survey: *N* = 1519 (students). For portfolio: *N* = 240 (students).	Purposive sampling. Survey (quantitative only), Portfolio data (subsample *N* = 60). Deductive content analysis.
Butani et al, 2021 [31]	Mixed‐methods, United States and Canada. Investigating extent and nature of EPA use in medical education, and barriers and facilitators to use.	Undergraduate medicine (paediatrics). *N* = 167 (directors, teaching faculty, administrators).	Census sampling. Web‐based survey (free text responses). Content analysis, iterative consensus process.
Duijn et al, 2018 [32]	Qualitative, Netherlands. Identifying which decision variables clinical teachers perceive as relevant when making entrustment decisions.	Undergraduate medicine/veterinary. *N* = 8 (medical teachers), *N* = 9 (veterinary teachers).	Purposive sampling. Focus groups. Open and axial coding (grounded theory).
Duijn et al, 2017 [33]	Qualitative, Netherlands. Identifying what students perceive as meaningful feedback required to prepare for an EPA.	Undergraduate medicine/veterinary. *N* = 32 (students).	Purposive sampling. Focus groups, nominal group technique. Open and axial coding (grounded theory).
Freedman et al, 2025 [34]	Mixed‐methods, United States. Exploring trainees' perceptions of the Goal orientated learner driven entrustment (GOLD‐E) tool.	Senior/clinical year veterinary trainees. For surveys: *N* = 30 (students). For focus groups: *N* = 18 (students).	Convenience sampling. Surveys (free text responses), focus groups. Inductive thematic analysis.
Gin et al, 2022 [35]	Mixed‐methods, United States. Identifying narrative feedback characteristics associated with entrustment decisions using machine learning.	Undergraduate medicine. *N* = 216 (students), *N* = 1455 (supervisors).	Convenience sampling. Observation (supervisor feedback, free text responses). Axial coding to interpret AI‐identified themes.
Lane et al, 2018 [36]	Qualitative, United States. Evaluating a tool prompting learner reflection to support assessment decisions.	Undergraduate medicine. *N* = 7 (students).	Purposive sampling. Structured reflection tool including preceptor feedback. Content analysis.
Lee et al, 2025 [37]	Mixed‐methods, Canada. Exploring students' perceptions of narrative feedback in EPA observations.	Undergraduate medicine. *N* = 35.	Non‐random, census‐style sampling. Online survey (free text responses). Inductive thematic analysis.
Li et al, 2024 [38]	Qualitative, Canada. Exploring sources of variation when assessors estimate learner's readiness for entrustment.	Undergraduate medicine (palliative care). *N* = 19 (faculty and learner assessors)	Non‐random, census‐style sampling. Small group debriefs (primary source of qualitative data), feedback, reflective memos. Constructivist grounded theory.
McDonald et al, 2025 [39]	Mixed methods, Australia. Evaluating how written feedback supports students' clinical learning.	Undergraduate medicine. *N* = 120	Purposive sampling. EPA forms (supervisor feedback, free text responses). Reflexive thematic analysis.
Palsson et al, 2024 [40]	Qualitative, Sweden. Investigating how supervisors experienced EPAs.	Undergraduate medicine. *N* = 10 (supervisors).	Purposive sampling. Semi‐structured interviews. Inductive content analysis.
Postmes et al, 2021 [41]	Qualitative, Netherlands. Exploring challenges clinical teachers face when using an entrustment‐supervision scale.	Undergraduate medicine. *N* = 120 (clinical teachers).	Purposive sampling. Semi‐structured interviews. Inductive open coding.

### 2.3 Risk of Bias

Across the studies, overall methodological quality was strong, with most studies rated as low concern, and only a small number showing moderate limitations (Table 3). Qualitative studies commonly demonstrated clear methods but were limited by minimal reflexivity and lack of explicit philosophical positioning. Mixed methods studies were generally robust, with concerns largely limited to survey‐related constraints, for example response rates and generalisability, rather than fundamental design flaws. Overall, the evidence base was judged to be methodologically credible, with limitations that were transparent, predictable, and not severe enough to undermine confidence in the synthesis.

**Table 3:** Quality appraisal summary.

Summary			
Papers	Tool used	Overall level of concern	Key limitations
Alexander et al, 2025 [27]	JBI^a^ qualitative	Low	Reflexivity offered is largely procedural. Limited discussion of research positionality.
Bradley et al, 2021 [28]	JBI qualitative	Low	Does not articulate an explicit philosophical stance. Researcher positionality is described but not explored culturally or theoretically.
Bramley et al, 2021 [29]	MMAT^b^	Moderate	Insufficient description of qualitative methods.
Bremer et al, 2025 [30]	MMAT	Low	Defensible mixed‐methods structure and interpretation, with limitations acknowledged.
Butani et al, 2021 [31]	MMAT	Low	Solid mixed‐methods survey, with limitations related to typical survey bias and anonymity constraints.
Duijn et al, 2018 [32]	JBI qualitative	Low	Does not articulate an explicit philosophical stance. Researcher backgrounds and relationships described but cultural and theoretical positioning not explicit.
Duijn et al, 2017 [33]	JBI qualitative	Low	Researcher backgrounds and relationships described but cultural and theoretical positioning not explicit.
Freedman et al, 2025 [34]	MMAT	Low	Robust mixed methods evaluation. Main weakness was generalisation.
Gin et al, 2022 [35]	MMAT	Low	Methodologically strong. Interpretative limits relate to assessment design and rating scale.
Lane et al, 2018 [36]	JBI qualitative	Moderate	Does not articulate an explicit philosophical stance. No reflexivity statement is provided, and researcher positionality is not addressed.
Lee et al, 2025 [37]	MMAT	Low	Well conduced mixed‐method survey. Minor issues relating to response rate and single site.
Li et al, 2024 [38]	JBI qualitative	Low	A methodologically exemplary qualitative study.
McDonald et al, 2025 [39]	MMAT	Low	Strong mixed‐methods study. Limitations relate to early implementation context and absence of verbal feedback data.
Palsson et al, 2024 [40]	JBI qualitative	Moderate	Does not articulate an explicit philosophical stance. No reflexivity statement is provided, and researcher positionality is minimally addressed.
Postmes et al, 2021 [41]	JBI qualitative	Moderate	Does not articulate an explicit philosophical stance. Researcher roles described, but reflexive positioning is not addressed.

a Joanna Briggs Institute (JBI) [22].

b Mixed‐Methods Assessment Tool (MMAT) [23].

### 2.4 Results of Individual Studies

**Figure 2:**
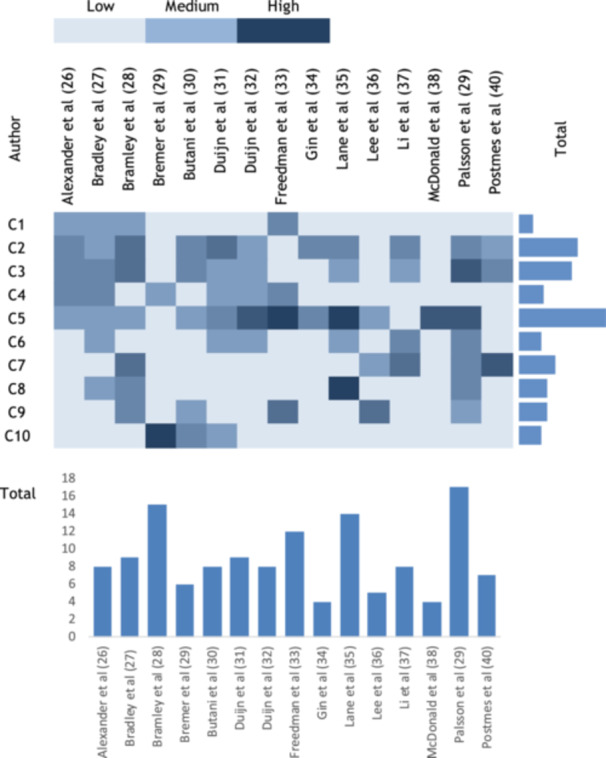
Heatmap of codes (C1‐C10) across studies.

First order constructs (participant quotes including academic, practice educator and learner), and second order interpretations (concepts and themes) were extracted to produce third order codes (Appendix 3). Each third order code is defined within a codebook (Appendix 4). A heatmap (Figure 2) illustrates how strongly each third order code (C1‐C10) appears across the included studies. The bar on the right of each row totals how frequently each code appeared across all studies, while the bar chart at the bottom of the figure summarises the total number of coded contributions per study. Table 4 selects a sample of first order participant quotations and second order concepts and themes, from the heatmap, to explicitly show the development of the third order codes.

**Table 4:** Development of third order codes.

Third order code	Illustrative first order quotes	Key second order concepts and themes
C1: Perceived task value and meaningful participation	*“It was something special, I mastered the challenge with joy.”* [27]. *“When people (educators) are forced to watch… they think a lot more critically about your performance”* [28].	Multi‐source input contributes to legitimacy and clarity around expectations [34].
C2: Supported autonomy	*“I trust the student to do this task with my supervision”* [31]. *“Do you think you can do it on your own?”* [32].	Concrete, specific reinforcement signals high trust [35].
C3: Supervision fit and conditions for safe performance	*“Less open to interpretation by your supervisor.”* [29]. *“Clear improvement…better structure of your assessments and your feedback”* [40].	Prospective scales demand that teachers move from “what happened” to “what is safe/appropriate next time,” a cognitive shift [41].
C4: Educator–Learner relationship and psychological safety	*“Difficult to overcome the fear… physicians here give one the peace of mind to do all this”* [27]. *“Part of a safe environment is also that you are able to mention having difficulties with a supervisor”* [33].	Trainees are uncertain about their role in the feedback relationship. They experience power imbalance, ambiguity, and fear of burdening clinicians [34].
C5: Actionable constructive feedback	*“Feedback needs to be focused on whether you can do this on your own or not”* [33]. *“I now understand a better way to give information”* [36].	Actionable, targeted advice provides “feed‑forward” strategy for improvement and supports future performance [39].
C6: Educator credibility, judgement and evidence	*“I tried to put aside his polite manner and really think about what information he gave and how he gave it.”* [38]. *“The most important thing is to know how a learner has been assessed…by multiple teachers… information from multiple sources”* [32].	EPA scales can create presumptive trust, but some supervisors require grounded trust (personal observation/relationship) and may resist relying on colleagues' ratings —especially with unfamiliar learners [40].
C7: Underlying assumptions and shortcuts in entrustment decisions	*“There were different ideas… some supervisors were expecting them to show every single competency with the patient…”* [29]. *“When they design the EPA to evaluate our supervision… mine will say ‘you jump in too often’”* [38].	Target levels can clarify expectations but also anchor ratings, compress variance, and act as a default when teachers feel uncertain or under‑informed [41].
C8: Learner agency, self‐assessment and regulation	*“It allows me to judge my competency level… what I need to work on and where I need to be by the end of placement.”* [29]. *“…students… focus more on the grade they get… which will perhaps cloud their memory of what was said…”* [40].	Reflection moves from recounting tasks to feed‑forward planning: identifying knowledge needs and specific next learning actions [36].
C9: Usability issues, workload pressure and box‐ticking	*“Finding the time within clinicians' schedule because they're busy…”* [34]. *“Wonder if they are present to force staff to provide adequate supervision… if I did not need an EPA… the staff would never have supervised me doing things.”* [37].	Students: usability and workload burdens. Supervisors: infrastructure, access, privacy issues. Confirm need for streamlined design and better technological support [29].
C10 Professional identity and readiness	*EPAs provide an “opportunity to longitudinally track student professional development”* [31]. *“I want to…trust a learner will execute this task the same way in the future”* [32].	Process‑focused feedback supports stronger professional identity formation than outcome‑focused feedback [30].

### 2.5 Results of Syntheses

**Figure 3:**
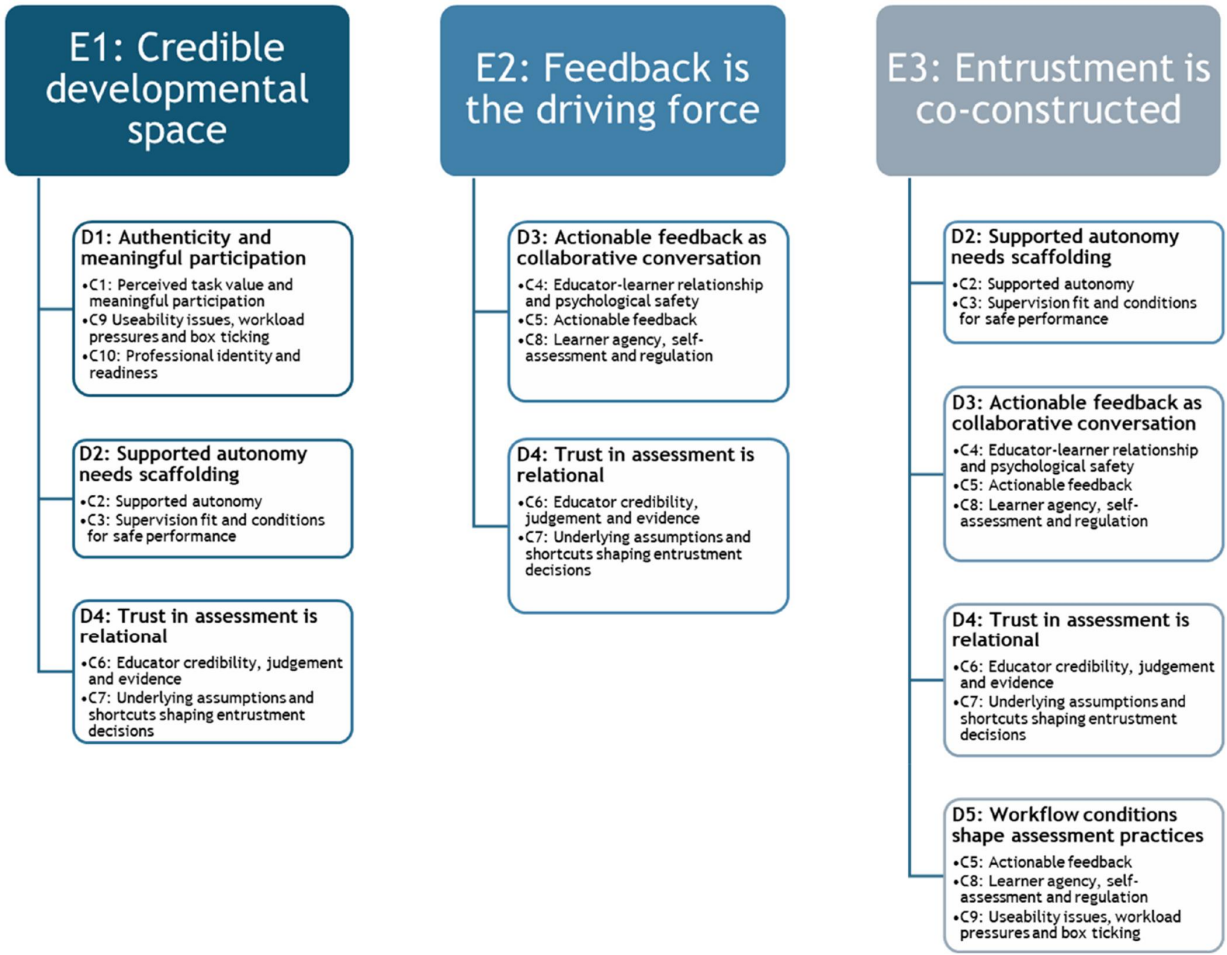
Thematic map of descriptive and analytical themes.

A thematic map (Figure 3) was constructed to illustrate the relationships between the three analytical themes (E1‐3), the related descriptive themes (D1‐5), and the third order codes (C1‐10). This map highlights the interconnected nature of developmental space, feedback processes, and the co‐construction of entrustment across learning environments.

## 3 Discussion

This qualitative systematic review synthesised 15 international studies to explore how entrustment is conceptualised and enacted in pre‐registration PBL. The findings reinforce the growing recognition that entrustment matters and is a central mechanism for structuring learner autonomy in complex, real‐world settings [10, 18, 21]. This complements the shift in healthcare education from competency‐based to capability‐orientated frameworks [4, 5, 6]. The descriptive themes (D1‐5) show how entrustment is conceptualised in real‐world PBL settings, while the analytical themes (E1‐3) show how entrustment is operationalised in education (Figure 3). All themes offer insight into implications for dietetic practice and future research.

### 3.1 Credible Developmental Space

Across the included studies, entrustment provided learners with meaningful participation in authentic work, reinforcing existing evidence that trust is central to workplace learning (D1, D4). The descriptive theme D1 (Authentic and meaningful participation) showed that learners experienced entrustment as active engagement in tasks that felt purposeful and legitimate, supported by supervisory structures that made participation feel safe (C1, C3). This finding strengthens long‐standing claims that EPAs help to create a more coherent learning pathway by linking supervision levels directly to real‐world PBL tasks [51, 52]. It also aligns with contemporary interpretations of safe professional practice in complex, uncertain environments [53, 54].

Looking deeper, entrustment functions not merely as an assessment judgement but as a credible developmental space, a structured environment where learners test, extend, and integrate capability with scaffolded supervision. This reframes entrustment from a tool for confirming competence to a pedagogical mechanism for fostering capability through participation, supervision, and reflection. The descriptive themes confirm that autonomy is scaffolded and negotiated (D2), rather than a binary judgement, aligning with critiques of competency frameworks that oversimplify progression into discrete steps [55, 56, 57].

This broader conceptualisation also highlights some limitations in common assumptions about EPAs. The review makes clear that the EPA framework does not automatically ensure authentic learning experiences. Learners described situations where EPA tasks felt tokenistic or artificially constructed, and where genuine participation relied on local workplace dynamics such as practice educator availability, workplace norms, and organisational pressures (D5). This analytical theme therefore challenges the idea that EPAs inherently deliver ‘authentic learning’, showing that authenticity is something constructed through relational, contextual, and organisational support rather than guaranteed by the framework alone. A recent study by Hennus et al [58] similarly found that success ‘depends on alignment with local context’.

Finally, recognising entrustment as developmental highlights how workflow conditions may distort entrustment decisions. Several studies indicated that supervisory decisions were shaped by staffing pressures or efficiency demands rather than learner capability (C9, D5). For example, practice educators described having to delay supervision decisions during busy shifts, limit observation to brief encounters, or prioritise service delivery over developmental opportunities. This challenges the assumption that entrustment‐based decisions are simply objective assessments of competence, emphasising instead that system‐level support is required to safeguard their developmental role.

### 3.2 Feedback Is the Driving Force

While feedback appeared throughout the included studies, the analysis reveals it as a far more foundational mechanism than often acknowledged. The descriptive theme D3 (Actionable feedback as a collaborative conversation) consistently demonstrated that learners relied on high quality narrative guidance to understand expectations, interpret supervision levels, and identify their next developmental steps (C5). This aligns with extensive evidence showing that effective, timely feedback is essential for learning and performance during PBL in pre‐registration health professions education [59].

However, this synthesis extends that understanding, showing feedback is the active ingredient that makes entrustment work. Entrustment decisions do not simply record the level of supervision, they produce information that learners must interpret and apply. Without corresponding dialogue and reflection, entrustment becomes a hollow administrative mechanism detached from the learner's educational experience.

This insight helps explain why learners sometimes became preoccupied with supervision levels as grades, rather than as developmental signals [42]. In systems where documentation dominates, feedback becomes under‐theorised and under‐emphasised. The analytical theme therefore challenges tool‐focused implementation of EPAs that over‐prioritise forms, or scales. For entrustment to function as a developmental process, practice educators and learners must engage in dialogic feedback that helps them make sense of supervisory decisions.

This theme also exposes a critical relational layer; learners described uncertainty about how to initiate feedback, fear of burdening practice educators, and sensitivity to power differentials (C4, C8). These dynamics illustrate why feedback cannot be treated as a technical add‐on to supervision but is fundamental to the relational environment in which entrustment occurs [60, 61]. Without attention to these relational factors, entrustment risks becoming bureaucratic rather than fostering capability development.

### 3.3 Entrustment Is Co‐Constructed

The third analytical theme helps deepen understanding of entrustment by showing that it is not simply a technical decision. The descriptive themes confirm the importance of trust (D4), the value of practice educators knowing their learners well (D1), and the influence of context on supervision decisions (D5). Here, the analytical synthesis goes a step further and explains why these factors matter for learning.

This review showed that entrustment involves far more than applying a supervision scale. Entrustment is a judgement shaped by practice educator confidence in the learner, their emotional reactions, their sense of risk, their familiarity with the learner, and the norms of their team or setting. The included studies revealed that practice educators often rely on tacit impressions, shared histories, and collective expectations (C6, C7). In this way, entrustment is always interpretative. Rather than viewing subjectivity as a flaw, this theme acknowledges subjectivity as an unavoidable and necessary part of assessing learners in complex environments.

Importantly, this synthesis also challenges the common assumption that entrustment is predominantly about granting independence. Learners did not necessarily interpret a less supervision as independent practice but rather as a form of relational autonomy, something negotiated within supportive relationships and shaped by local constraints. This helps explain why some learners felt trusted even with more supervision, while others felt restricted despite receiving higher entrustment ratings. It illustrates how much relational interpretation is involved in making supervision scales meaningful in practice.

Context adds another layer of complexity. Practice educators reported difficulty trusting colleagues' ratings, uneasy about making feed‐forward judgements around entrustment, and tension between prioritising safety and creating learning opportunities (C9, C6). Organisational pressures such as workload, documentation, and fragmented observation (D5) further shaped entrustment decisions [62]. These findings show that entrustment reflects the wider practice environment, not just an individual learner's capability.

Overall, this analytical theme reframes entrustment as a relational judgement embedded in context. To support good entrustment decisions, educational structures need to encourage a shared understanding, opportunities for multiple practice educators to contribute, continuity of supervision, and protected time for practice educators and learners to interpret decisions together.

### 3.4 Implications for UK Dietetic Profession

The move toward a UK‐wide dietetic common assessment tool provides a timely opportunity to align PBL with the broader shift in UK health professions education toward capability‐oriented, cross setting assessment. This builds on existing dietetics literature, which has highlighted challenges in translating competency frameworks into practice and the value of structured tools such as e‐portfolios and EPAs in supporting workplace‐based assessment [14, 15, 38]. This synthesis highlights several implications that can guide implementation.

1. Entrustment can strengthen developmental assessment in dietetics.
  This review shows that entrustment offers a structured, relational way to support learner autonomy and capability development. Used thoughtfully, it can help practice educators make clearer, more defensible decisions about learner progression while maintaining a focus on growth rather than compliance.
2. Narrative, future‐focused feedback must remain central.
  Learners rely on high quality, dialogic feedback to interpret supervision levels and understand what capability looks like in practice. Dietetic assessment tools will need to continue to prioritise judgement informed by holistic feedback, ensuring that supervision levels are accompanied by actionable feedback that helps learners interpret, make sense of, and act on the decision.
3. Practice educator preparation is essential but should remain practical and manageable.
  The findings show that entrustment requires a cognitive shift, from checking competency to making contextual judgements. Supporting practice educators to do this confidently does not require complex new tools, but rather opportunities to discuss examples, calibrate decisions, and understand what developmental entrustment looks like in dietetic PBL settings.
4. A light touch, EPA approach could offer coherence without overwhelming practice educators.
  Although EPAs are not yet operationalised in UK dietetics, the profession can benefit from adopting simple entrustment aligned tasks. Using one meaningful EPA‐like activity per capability may offer the right balance, clear enough to support shared expectations across settings, but simple enough to avoid additional burden. This approach would help bridge capability statements and real‐world assessment decisions without introducing a large new classification system.
5. System‐level design must recognise the realities of PBL settings.

The review makes clear that entrustment decisions are shaped by workload, documentation requirements, practice educator availability, and fragmented observation. Supporting the implementation of assessment processes that are streamlined, easy to use, and attentive to relational dynamics will be essential to ensuring that entrustment supports learning rather than becoming another bureaucratic demand.

Overall, these implications suggest that entrustment can strengthen UK dietetic education when implemented in a relational, proportionate, and capability‐aligned way. By explicitly linking to and extending an emerging dietetics evidence base on EPA use, feedback and competency translation, this review provides a conceptual and practical bridge between existing dietetic assessment approaches and more integrated, entrustment‐informed models of PBL. This is consistent with wider health professions education evidence on entrustment and workplace‐based assessment [63, 64].

While this review highlights the potential of entrustment to strengthen assessment, these implications must be considered alongside practical challenges. Implementation in dietetic PBL may be constrained by workload pressures and competing service demands which limit the time available for meaningful feedback. In the UK dietetic context, similar pressures are likely to influence adoption, particularly given increasing PBL capacity demands and practice educator workload.

Practice educators may also require support to develop confidence in making holistic entrustment judgements. A proportionate, streamlined approach that integrates entrustment within existing processes may help mitigate these challenges while preserving its developmental benefits.

### 3.5 Global Implications for Dietetics Education

This review has important implications for dietetics education both in the UK and internationally. Entrustment was consistently described as a relational judgement, shaped by trust, familiarity, workplace culture and perceived risk, rather than a technical rating of task completion. Common challenges across international PBL systems such as time pressure, fragmented opportunities for observation and variable practice educator availability, affect how these judgements are made. Without high quality, dialogic feedback, entrustment risks becoming a procedural requirement rather than a meaningful support for learner development.

The synthesis provides a foundation for designing practice educator training that strengthens relational supervision skills and helps practice educators use entrustment as a mechanism for building capability rather than checking competence. The findings also contribute to global conversations about programmatic assessment, where rich, triangulated narrative evidence is increasingly used to inform high stakes decisions and to differentiate day‐to‐day workplace feedback from summative academic decision‐making. These programmatic assessment approaches are increasingly reflected in health professions education, with growing implementation within dietetics programmes [65, 66].

Finally, the review highlights the need for international comparative research, particularly across healthcare professions and regulatory systems with differing levels of risk and complexity. As many countries adopt more integrated, cross setting models of PBL, the conceptual model emerging from this synthesis, entrustment as a credible developmental space co‐constructed through trust and activated through high quality feedback, offers a shared framework that may support global alignment in supervision and assessment practices.

### 3.6 Implications for Future Research

Future research in dietetic education should build on these findings by examining how entrustment‐based decisions function within UK PBL specifically, as the existing evidence originates predominantly from medical education. While the underlying principles of workplace learning and entrustment are shared across health professions, differences in scope of practice, supervision models, and PBL structures may influence how these approaches are operationalised in dietetics. Studies are needed to understand how dietetic practice educators interpret supervision levels, negotiate risk, and balance safety with developmental opportunity in real PBL settings.

Research should also explore the feasibility and impact of a simplified EPA approach in dietetics. Evaluating whether one EPA‐like task per capability improves clarity, consistency, and practice educator confidence would help identify the right level of detail for the profession. This is particularly relevant in a context where simplicity and workload management are key priorities.

Given the relational nature of entrustment, further work should investigate how practice educators and learners engage in dialogic feedback and sense‐making conversations. Understanding the conditions that enable or constrain these interactions, including workload, supervision models, and organisational culture, will be crucial for implementing assessment processes that genuinely support capability development.

Finally, future research could examine the role of ‘safe uncertainty’ in dietetic education. Understanding how practice educators make judgements in situations that are complex, ambiguous, or only partially observable may shed light on how to better support them in making developmental, prospective entrustment decisions across varied PBL settings.

## 4 Limitations

This review has several limitations. The evidence base was dominated by undergraduate medical education, with relatively few studies from dietetics or allied health, which may limit direct transferability to the UK dietetic context. Although the majority of studies demonstrated strong methodological rigour, some qualitative studies lacked reflexivity or explicit philosophical positioning, elements that can limit interpretive depth and transparency [45, 49, 50]. Entrustment and EPAs were also implemented inconsistently across countries and professions, reflecting heterogeneity in PBL structures, assessment cultures, and regulatory expectations.

Additionally, this review included only English‐language publications, raising the possibility of language or publication bias despite targeted grey literature searching [30]. Reflexive processes and explicit philosophical positioning helped mitigate researcher bias, while grey literature searching, citation chaining, and the use of a transparent exclusion list strengthened the comprehensiveness of the search strategy [34]. Nonetheless, the findings should be viewed in light of these limitations.

## 5 Conclusion

Entrustment in pre‐registration PBL emerges from this review as a dynamic, relational, and context dependent process that supports learner progression, structures supervision, and fosters capability development. It is more than a technical judgement; it is shaped by trust, communication, contextual awareness, and the quality of relationships. These features align closely with the needs of contemporary UK healthcare education, where learners must develop the ability to practise safely and adaptively within complexity and uncertainty.

For the UK dietetic profession, entrustment informed assessment approaches support a sector moves toward capability based, cross setting PBL and a national common assessment tool. Realising this potential will require attention to the relational and contextual foundations of entrustment, including practice educator‐learner dialogue, psychological safety, and organisational support. A proportionate, light touch approach that uses a small number of well‐chosen EPA aligned tasks, supported by succinct, high quality narrative feedback, may improve consistency and developmental value without increasing burden for practice educators.

Taken together, this review suggests that entrustment can meaningfully strengthen the coherence, equity, and educational richness of PBL in UK dietetic education when implemented thoughtfully. By keeping relational judgement, shared sensemaking, and capability development at the heart of assessment, the profession is well placed to adopt an approach that is both developmentally robust and responsive to the realities of contemporary practice.

## Author Contributions

Ruth Boocock was responsible for the study conception and design. Ruth Boocock was responsible for the literature search. Ruth Boocock was responsible for data collection. Ruth Boocock, Cecile Jones and Amelia A. Lake were responsible for analysis and interpretation. Ruth Boocock was responsible for the integrity of data analysis. Ruth Boocock was responsible for the writing the article. Cecile Jones and Amelia A. Lake were responsible for critical revision of the article. Ruth Boocock, Cecile Jones and Amelia A. Lake approved the final version of the article submitted for publication.

## Funding

The authors have nothing to report.

## Ethics Statement

This review used only published sources of data. Ethical review by a Research Ethics Committee was not required.

## Conflicts of Interest

The authors RB and CJ declare no conflicts of interest. AL is Deputy Director of Fuse the Centre for Translational Research in Public Health and is Professor of Public Health Nutrition at Teesside University. She sits on the scientific committee of the British Nutrition Foundation and is an executive for Nutrition North (Northern Health Science Alliance).

## Supporting information

Supporting File S1

Supporting File S2

## Data Availability

The data that supports the findings of this study are available in the supplementary material of this article.
