# Supplementary material for: A Qualitative Systematic Review of the Role of Entrustment in Pre‐Registration Healthcare Practice‐Based Learning: Implications for Dietetics Education
Source: J Hum Nutr Diet. 2026 Jun 10;39(3):e70287. doi: 10.1111/jhn.70287 (PMC13253917; doi:10.1111/jhn.70287)
Supplement: Supplementary file 1 — Supporting File S1 [file JHN-39-0-s001.docx]

## Contents

[Contents 1](#_Toc222222389)

[Appendix 1: Full search strategies 1](#_Toc222222390)

[Search strategy in evidence-based framework (PEO) 1](#_Toc222222391)

[Ebsco-based database search 2](#_Toc222222392)

[Embase search 3](#_Toc222222393)

## Appendix 1: Full search strategies

### Search strategy in evidence-based framework (PEO)

| P (Population)  (P^1^ **AND** P^2^) | | E (exposure)  (E) | O (outcome)  (O) |
| --- | --- | --- | --- |
| Healthcare Educat* | Practice educator* | Entrustment-based “Decision Making” | Use |
| Medical educat* | Clinical educator* | “Entrustment based” “Decision Making” | outcome* |
| “Allied health” educat* | Mentor | “Entrustment based” supervision | Attitud* |
|  | Supervisor | Entrustment-based supervision | perceiv* |
|  | Learner | Entrustment-based assessment | Efficacy |
|  | Apprentice | “Entrustment based” assessment | performance |
|  | Stakeholder | Entrustable professional activit* | Views |
|  | Pre-registration | Entrustable professional assessment | perception* |
|  | Postgraduate | EPAs | Experience |
|  | Teacher |  | Implementation |
|  | Practice teacher |  | Consequenc* |
|  | Clinical teacher |  | effect* |
|  | undergraduate |  | impact* |
|  | graduate |  | implication* |
|  | educator* |  | perspective* |
|  |  |  | thought* |
|  |  |  | opinion* |
|  |  |  | judg* |
|  |  |  | expectation* |
|  |  |  | belief* |
|  |  |  | Readiness N2 practice |
|  |  |  | behavio* |

### Ebsco-based database search

|  | **Option 3** |  |
| --- | --- | --- |
| **#** | **Query** | **Limiters/Expanders** |
| S55 | S21 AND S31 AND S54 | Search modes - Proximity |
| S54 | S32 OR S33 OR S34 OR S35 OR S36 OR S37 OR S38 OR S39 OR S40 OR S41 OR S42 OR S43 OR S44 OR S45 OR S46 OR S47 OR S48 OR S49 OR S50 OR S51 OR S52 OR S53 | Search modes - Proximity |
| S53 | judg* | Search modes - Proximity |
| S52 | expectation* | Search modes - Proximity |
| S51 | thought* | Search modes - Proximity |
| S50 | Use | Search modes - Proximity |
| S49 | outcome* | Search modes - Proximity |
| S48 | impact* | Search modes - Proximity |
| S47 | effect* | Search modes - Proximity |
| S46 | perception* | Search modes - Proximity |
| S45 | performance | Search modes - Proximity |
| S44 | Views | Search modes - Proximity |
| S43 | opinion* | Search modes - Proximity |
| S42 | Consequenc* | Search modes - Proximity |
| S41 | perceiv* | Search modes - Proximity |
| S40 | implication* | Search modes - Proximity |
| S39 | perspective* | Search modes - Proximity |
| S38 | Experience | Search modes - Proximity |
| S37 | Implementation | Search modes - Proximity |
| S36 | Readiness N2 practice | Search modes - Proximity |
| S35 | Attitud* | Search modes - Proximity |
| S34 | behavio* | Search modes - Proximity |
| S33 | Efficacy | Search modes - Proximity |
| S32 | belief* | Search modes - Proximity |
| S31 | S22 OR S23 OR S24 OR S25 OR S26 OR S27 OR S28 OR S29 OR S30 | Search modes - Proximity |
| S30 | EPAs | Search modes - Proximity |
| S29 | “Entrustment based” “Decision Making” | Search modes - Proximity |
| S28 | Entrustment-based supervision | Search modes - Proximity |
| S27 | Entrustable professional assessment | Search modes - Proximity |
| S26 | “Entrustment based” assessment | Search modes - Proximity |
| S25 | Entrustment-based assessment | Search modes - Proximity |
| S24 | Entrustable professional activit* | Search modes - Proximity |
| S23 | Entrustment-based “Decision Making” | Search modes - Proximity |
| S22 | “Entrustment based” supervision | Search modes - Proximity |
| S21 | S4 AND S20 | Search modes - Proximity |
| S20 | S5 OR S6 OR S7 OR S8 OR S9 OR S10 OR S11 OR S12 OR S13 OR S14 OR S15 OR S16 OR S17 OR S18 OR S19 | Search modes - Proximity |
| S19 | Practice teacher | Search modes - Proximity |
| S18 | Practice educator* | Search modes - Proximity |
| S17 | Clinical educator* | Search modes - Proximity |
| S16 | Mentor | Search modes - Proximity |
| S15 | Stakeholder | Search modes - Proximity |
| S14 | Postgraduate | Search modes - Proximity |
| S13 | Graduate | Search modes - Proximity |
| S12 | Teacher | Search modes - Proximity |
| S11 | Undergraduate | Search modes - Proximity |
| S10 | educator* | Search modes - Proximity |
| S9 | Clinical teacher | Search modes - Proximity |
| S8 | Supervisor | Search modes - Proximity |
| S7 | Learner | Search modes - Proximity |
| S6 | Pre-registration | Search modes - Proximity |
| S5 | Apprentice | Search modes - Proximity |
| S4 | S1 OR S2 OR S3 | Search modes - Proximity |
| S3 | “Allied health” educat* | Search modes - Proximity |
| S2 | Medical educat* | Search modes - Proximity |
| S1 | Healthcare Educat* | Search modes - Proximity |

### Embase search

|  | Option 3 |
| --- | --- |
| 1. | Healthcare Educat*.mp. [mp=title, abstract, heading word, drug trade name, original title, device manufacturer, drug manufacturer, device trade name, keyword heading word, floating subheading word, candidate term word] |
| 2. | Medical educat*.mp. [mp=title, abstract, heading word, drug trade name, original title, device manufacturer, drug manufacturer, device trade name, keyword heading word, floating subheading word, candidate term word] |
| 3. | "Allied health educat*".mp. [mp=title, abstract, heading word, drug trade name, original title, device manufacturer, drug manufacturer, device trade name, keyword heading word, floating subheading word, candidate term word] |
| 4. | 1 or 2 or 3 |
| 5. | Practice educator*.mp. [mp=title, abstract, heading word, drug trade name, original title, device manufacturer, drug manufacturer, device trade name, keyword heading word, floating subheading word, candidate term word] |
| 6. | Clinical educator*.mp. [mp=title, abstract, heading word, drug trade name, original title, device manufacturer, drug manufacturer, device trade name, keyword heading word, floating subheading word, candidate term word] |
| 7. | Mentor*.mp. [mp=title, abstract, heading word, drug trade name, original title, device manufacturer, drug manufacturer, device trade name, keyword heading word, floating subheading word, candidate term word] |
| 8. | Supervisor*.mp. [mp=title, abstract, heading word, drug trade name, original title, device manufacturer, drug manufacturer, device trade name, keyword heading word, floating subheading word, candidate term word] |
| 9. | Learner*.mp. [mp=title, abstract, heading word, drug trade name, original title, device manufacturer, drug manufacturer, device trade name, keyword heading word, floating subheading word, candidate term word] |
| 10. | Apprentice*.mp. [mp=title, abstract, heading word, drug trade name, original title, device manufacturer, drug manufacturer, device trade name, keyword heading word, floating subheading word, candidate term word] |
| 11. | Stakeholder*.mp. [mp=title, abstract, heading word, drug trade name, original title, device manufacturer, drug manufacturer, device trade name, keyword heading word, floating subheading word, candidate term word] |
| 12. | Pre-registration*.mp. [mp=title, abstract, heading word, drug trade name, original title, device manufacturer, drug manufacturer, device trade name, keyword heading word, floating subheading word, candidate term word] |
| 13. | Postgraduate*.mp. [mp=title, abstract, heading word, drug trade name, original title, device manufacturer, drug manufacturer, device trade name, keyword heading word, floating subheading word, candidate term word] |
| 14. | Teacher*.mp. [mp=title, abstract, heading word, drug trade name, original title, device manufacturer, drug manufacturer, device trade name, keyword heading word, floating subheading word, candidate term word] |
| 15. | Practice teacher*.mp. [mp=title, abstract, heading word, drug trade name, original title, device manufacturer, drug manufacturer, device trade name, keyword heading word, floating subheading word, candidate term word] |
| 16. | Clinical teacher*.mp. [mp=title, abstract, heading word, drug trade name, original title, device manufacturer, drug manufacturer, device trade name, keyword heading word, floating subheading word, candidate term word] |
| 17. | Undergraduate*.mp. [mp=title, abstract, heading word, drug trade name, original title, device manufacturer, drug manufacturer, device trade name, keyword heading word, floating subheading word, candidate term word] |
| 18. | Graduate*.mp. [mp=title, abstract, heading word, drug trade name, original title, device manufacturer, drug manufacturer, device trade name, keyword heading word, floating subheading word, candidate term word] |
| 19. | educator*.mp. [mp=title, abstract, heading word, drug trade name, original title, device manufacturer, drug manufacturer, device trade name, keyword heading word, floating subheading word, candidate term word] |
| 20. | 5 or 6 or 7 or 8 or 9 or 10 or 11 or 12 or 13 or 14 or 15 or 16 or 17 or 18 or 19 |
| 21. | 4 and 20 |
| 22. | "Entrustment-based Decision Making".mp. [mp=title, abstract, heading word, drug trade name, original title, device manufacturer, drug manufacturer, device trade name, keyword heading word, floating subheading word, candidate term word] |
| 23. | "Entrustment based Decision Making".mp. [mp=title, abstract, heading word, drug trade name, original title, device manufacturer, drug manufacturer, device trade name, keyword heading word, floating subheading word, candidate term word] |
| 24. | "Entrustment based supervision".mp. [mp=title, abstract, heading word, drug trade name, original title, device manufacturer, drug manufacturer, device trade name, keyword heading word, floating subheading word, candidate term word] |
| 25. | Entrustment-based supervision.mp. [mp=title, abstract, heading word, drug trade name, original title, device manufacturer, drug manufacturer, device trade name, keyword heading word, floating subheading word, candidate term word] |
| 26. | Entrustment-based assessment*.mp. [mp=title, abstract, heading word, drug trade name, original title, device manufacturer, drug manufacturer, device trade name, keyword heading word, floating subheading word, candidate term word] |
| 27. | "Entrustment based assessment*".mp. [mp=title, abstract, heading word, drug trade name, original title, device manufacturer, drug manufacturer, device trade name, keyword heading word, floating subheading word, candidate term word] |
| 28. | Entrustable professional activit*.mp. [mp=title, abstract, heading word, drug trade name, original title, device manufacturer, drug manufacturer, device trade name, keyword heading word, floating subheading word, candidate term word] |
| 29. | Entrustable professional assessment*.mp. [mp=title, abstract, heading word, drug trade name, original title, device manufacturer, drug manufacturer, device trade name, keyword heading word, floating subheading word, candidate term word] |
| 30. | EPAs.mp. [mp=title, abstract, heading word, drug trade name, original title, device manufacturer, drug manufacturer, device trade name, keyword heading word, floating subheading word, candidate term word] |
| 31. | 22 or 23 or 24 or 25 or 26 or 27 or 28 or 29 or 30 |
| 32. | Use*.mp. [mp=title, abstract, heading word, drug trade name, original title, device manufacturer, drug manufacturer, device trade name, keyword heading word, floating subheading word, candidate term word] |
| 33. | outcome*.mp. [mp=title, abstract, heading word, drug trade name, original title, device manufacturer, drug manufacturer, device trade name, keyword heading word, floating subheading word, candidate term word] |
| 34. | Attitud*.mp. [mp=title, abstract, heading word, drug trade name, original title, device manufacturer, drug manufacturer, device trade name, keyword heading word, floating subheading word, candidate term word] |
| 35. | perceiv*.mp. [mp=title, abstract, heading word, drug trade name, original title, device manufacturer, drug manufacturer, device trade name, keyword heading word, floating subheading word, candidate term word] |
| 36. | Efficacy.mp. [mp=title, abstract, heading word, drug trade name, original title, device manufacturer, drug manufacturer, device trade name, keyword heading word, floating subheading word, candidate term word] |
| 37. | performance*.mp. [mp=title, abstract, heading word, drug trade name, original title, device manufacturer, drug manufacturer, device trade name, keyword heading word, floating subheading word, candidate term word] |
| 38. | Views.mp. [mp=title, abstract, heading word, drug trade name, original title, device manufacturer, drug manufacturer, device trade name, keyword heading word, floating subheading word, candidate term word] |
| 39. | perception*.mp. [mp=title, abstract, heading word, drug trade name, original title, device manufacturer, drug manufacturer, device trade name, keyword heading word, floating subheading word, candidate term word] |
| 40. | Experience*.mp. [mp=title, abstract, heading word, drug trade name, original title, device manufacturer, drug manufacturer, device trade name, keyword heading word, floating subheading word, candidate term word] |
| 41. | Implementation.mp. [mp=title, abstract, heading word, drug trade name, original title, device manufacturer, drug manufacturer, device trade name, keyword heading word, floating subheading word, candidate term word] |
| 42. | Consequenc*.mp. [mp=title, abstract, heading word, drug trade name, original title, device manufacturer, drug manufacturer, device trade name, keyword heading word, floating subheading word, candidate term word] |
| 43. | effect*.mp. [mp=title, abstract, heading word, drug trade name, original title, device manufacturer, drug manufacturer, device trade name, keyword heading word, floating subheading word, candidate term word] |
| 44. | impact*.mp. [mp=title, abstract, heading word, drug trade name, original title, device manufacturer, drug manufacturer, device trade name, keyword heading word, floating subheading word, candidate term word] |
| 45. | implication*.mp. [mp=title, abstract, heading word, drug trade name, original title, device manufacturer, drug manufacturer, device trade name, keyword heading word, floating subheading word, candidate term word] |
| 46. | perspective*.mp. [mp=title, abstract, heading word, drug trade name, original title, device manufacturer, drug manufacturer, device trade name, keyword heading word, floating subheading word, candidate term word] |
| 47. | thought*.mp. [mp=title, abstract, heading word, drug trade name, original title, device manufacturer, drug manufacturer, device trade name, keyword heading word, floating subheading word, candidate term word] |
| 48. | opinion*.mp. [mp=title, abstract, heading word, drug trade name, original title, device manufacturer, drug manufacturer, device trade name, keyword heading word, floating subheading word, candidate term word] |
| 49. | judg*.mp. [mp=title, abstract, heading word, drug trade name, original title, device manufacturer, drug manufacturer, device trade name, keyword heading word, floating subheading word, candidate term word] |
| 50. | expectation*.mp. [mp=title, abstract, heading word, drug trade name, original title, device manufacturer, drug manufacturer, device trade name, keyword heading word, floating subheading word, candidate term word] |
| 51. | belief*.mp. [mp=title, abstract, heading word, drug trade name, original title, device manufacturer, drug manufacturer, device trade name, keyword heading word, floating subheading word, candidate term word] |
| 52. | (Readiness adj2 practice).mp. [mp=title, abstract, heading word, drug trade name, original title, device manufacturer, drug manufacturer, device trade name, keyword heading word, floating subheading word, candidate term word] |
| 53. | behavio*.mp. [mp=title, abstract, heading word, drug trade name, original title, device manufacturer, drug manufacturer, device trade name, keyword heading word, floating subheading word, candidate term word] |
| 54. | 32 or 33 or 34 or 35 or 36 or 37 or 38 or 39 or 40 or 41 or 42 or 43 or 44 or 45 or 46 or 47 or 48 or 49 or 50 or 51 or 52 or 53 |
| 55. | 21 and 31 and 54 |
